# The effect of age on longitudinal measures of beta cell function and insulin sensitivity during the progression of early stage type 1 diabetes

**DOI:** 10.1007/s00125-022-05836-w

**Published:** 2022-12-02

**Authors:** Ele Ferrannini, Andrea Mari, Gabriela S. F. Monaco, Jay S. Skyler, Carmella Evans-Molina

**Affiliations:** 1 CNR Institute of Clinical Physiology, Pisa, Italy; 2 CNR Institute of Neuroscience, Padua, Italy; 3 Department of Pediatrics, Indiana University School of Medicine, Indianapolis, IN USA; 4 The Herman B Wells Center for Pediatric Research, Indiana University School of Medicine, Indianapolis, IN USA; 5 Center for Diabetes and Metabolic Diseases, Indiana University School of Medicine, Indianapolis, IN USA; 6 Diabetes Research Institute, University of Miami, Miami, FL USA; 7 Roudebush VA Medical Center, Indianapolis, IN USA

**Keywords:** Age, Beta cell glucose sensitivity, C-peptide, Type 1 diabetes

## Abstract

**Aim/hypothesis:**

The risk of progressing from autoantibody positivity to type 1 diabetes is inversely related to age. Separately, whether age influences patterns of C-peptide loss or changes in insulin sensitivity in autoantibody-positive individuals who progress to stage 3 type 1 diabetes is unclear.

**Methods:**

Beta cell function and insulin sensitivity were determined by modelling of OGTTs performed in 658 autoantibody-positive participants followed longitudinally in the Diabetes Prevention Trial–Type 1 (DPT-1). In this secondary analysis of DPT-1 data, time trajectories of beta cell function and insulin sensitivity were analysed in participants who progressed to type 1 diabetes (progressors) to address the impact of age on patterns of metabolic progression to diabetes.

**Results:**

Among the entire DPT-1 cohort, the highest discriminant age for type 1 diabetes risk was 14 years, with participants aged <14 years being twice as likely to progress to type 1 diabetes as those aged ≥14 years. At study entry, beta cell glucose sensitivity was impaired to a similar extent in progressors aged <14 years and progressors aged ≥14 years. From study entry to stage 3 type 1 diabetes onset, beta cell glucose sensitivity and insulin sensitivity declined in both progressor groups. However, there were no significant differences in the yearly rate of decline in either glucose sensitivity (−13.7 [21.2] vs −11.9 [21.5] pmol min^−1^ m^−2^ [mmol/l]^−1^, median [IQR], *p*=0.52) or insulin sensitivity (−22 [37] vs −14 [40] ml min^−1^ m^−2^, median [IQR], *p*=0.07) between progressors aged <14 years and progressors aged ≥14 years.

**Conclusions/interpretation:**

Our data indicate that during progression to stage 3 type 1 diabetes, rates of change in declining glucose and insulin sensitivity are not significantly different between progressors aged <14 years and progressors aged ≥14 years. These data suggest there is a predictable course of declining metabolic function during the progression to type 1 diabetes that is not influenced by age.

**Graphical abstract:**

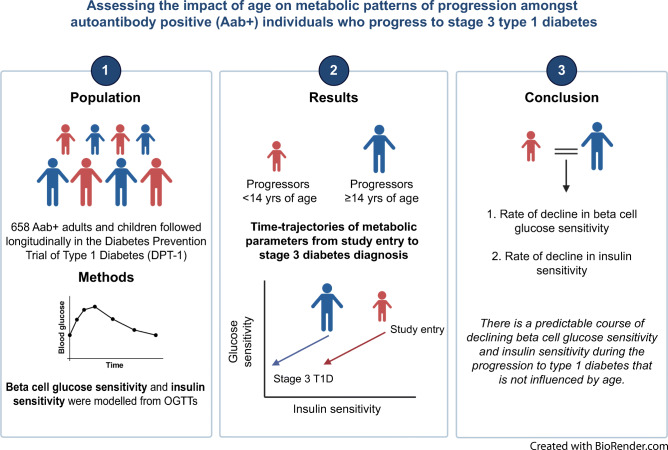

**Supplementary Information:**

The online version contains peer-reviewed but unedited supplementary material available at 10.1007/s00125-022-05836-w.

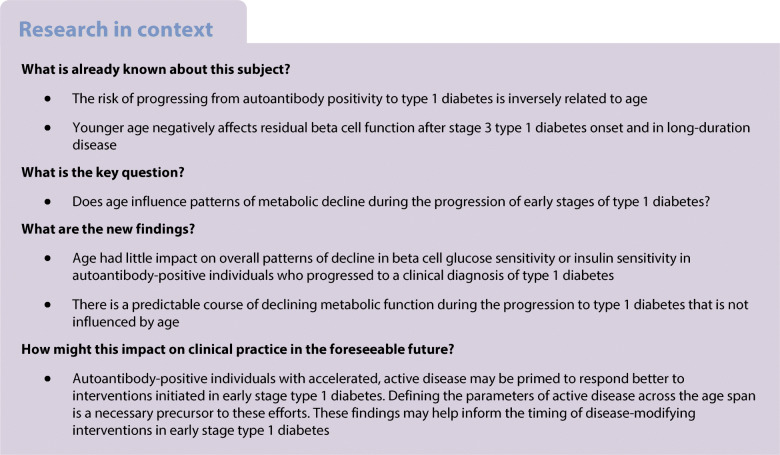



## Introduction

Type 1 diabetes results from immune-mediated destruction of the pancreatic beta cells, resulting in chronic hyperglycaemia, vascular complications and reduced life expectancy [1]. Stage 1 type 1 diabetes is defined as the presence of two of more islet autoantibodies and normal glucose tolerance. Stage 2 disease is defined as the presence of multiple islet autoantibodies and dysglycaemia, and stage 3 diabetes is marked by the development of hyperglycaemia that exceeds the ADA thresholds for clinical diagnosis [2]. Data from birth cohorts indicate that more than 70% of individuals with multiple autoantibodies will develop type 1 diabetes over 10 years of follow-up and nearly 85% will develop diabetes within 15 years of follow-up [3]. However, even within this staging framework, the timing of diabetes development can vary widely between individuals and is influenced by a number of demographic features. The age of autoantibody ascertainment is one of the most important factors driving disease-related heterogeneity, and it is uniformly accepted that age is inversely associated with the risk of developing type 1 diabetes among autoantibody-positive individuals [4]. Additionally, some but not all studies suggest that younger individuals manifest a more rapid loss of C-peptide following onset of stage 3 type 1 diabetes [5–8]. Consistent with these findings, measurable serum C-peptide and histological evidence of residual insulin-containing islets in long-duration disease are less likely to be observed in individuals with a younger age of onset [9–12]. While these data suggest potential differences in metabolic patterns between adults and children at or after stage 3 disease onset, whether age influences patterns of C-peptide loss or changes in insulin sensitivity prior to stage 3 onset has not been fully addressed.

Understanding whether there are differences in metabolic patterns of progression among individuals with autoantibody positivity across a diverse range of ages may have important implications for the timing and administration of immune modulatory interventions. To this end, we analysed data from longitudinal OGTTs performed in adult and youth participants in the Diabetes Prevention Trial–Type 1 (DPT-1), using previously validated models that provide estimates of both beta cell function and insulin sensitivity [13–17]. The DPT-1 enrolled multiple autoantibody-positive individuals and tested the efficacy of either parenteral or oral insulin in delaying the progression to stage 3 disease. While neither intervention impacted the development of diabetes, the DPT-1 dataset has provided a rich resource for understanding the natural history of type 1 diabetes [18, 19]. We tested how model-derived measures can aid in type 1 diabetes risk prediction and assessed the impact of age on metabolic patterns of progression prior to the onset of stage 3 type 1 diabetes.

## Methods

### DPT-1 study procedures

This manuscript presents a secondary analysis of data from the DPT-1 study (ClinicalTrials.gov registration no. NCT00004984). DPT-1 screened 103,391 relatives of individuals with type 1 diabetes, and study procedures have been described in detail [18, 19]. In brief, individuals eligible for screening had a first-degree relative with type 1 diabetes and were between 3 and 45 years of age or had a second-degree relative with type 1 diabetes and were between 3 and 20 years of age. The 3483 relatives who were found to be islet cell antibody (ICA) positive underwent metabolic, genetic and immunological staging to determine a 5 year risk of diabetes. Staging consisted of ICA confirmation, HLA-DQ typing, determination of insulin autoantibodies, and OGTT and IVGTT. Those considered to have >50% 5 year risk were eligible for entry into the parenteral insulin trial, while individuals with a 5 year risk of 25–50% were eligible for the oral insulin trial. Age was not considered when assigning risk categories. The primary outcome for both trials was progression to diabetes, and neither intervention showed a statistically significant difference between the treatment arms. Of the original 711 participants, complete C-peptide data for the present study were available for 658 individuals; for this reason, the HR and 95% CIs reported here differ slightly from those of the original publications [18, 19].

### Study protocol

The OGTT was performed using a dose of glucose of 1.75 g/kg body weight (maximum 75 g). After the participants had fasted overnight, blood samples were obtained through indwelling catheters for plasma glucose and serum C-peptide measurements in the fasting state and 30, 60, 90 and 120 min later. All participants (*n*=658) received an OGTT at baseline and at diabetes diagnosis or study end. In addition, whenever possible, the OGTT was repeated approximately every 6 months between baseline and diagnosis or study end (total of 4152 tests). Each study participant received a mean of 6.3 OGTTs [18, 19].

### Diagnosis of diabetes

Diabetes was diagnosed if the fasting glucose was ≥7.0 mmol/l or the 2 h glucose was ≥11.1 mmol/l, with confirmation by either an elevated fasting or 2 h glucose level at a special follow-up visit or a random plasma glucose ≥11.1 mmol/l accompanied by symptoms of polyuria, polydipsia or weight loss. Impaired glucose tolerance (IGT) was defined as a fasting glucose <7.0 mmol/l and a 2 h glucose between 7.8 and 11.1 mmol/l [18, 19].

### Analytical measurements

Plasma glucose was measured by the glucose oxidase method. Serum C-peptide was determined by RIA as previously described, with an interassay CV of 6.9% in a reference pool with relatively high values and 7.8% in a reference pool with relatively low values [18, 19].

### Data analysis

Modelling methods are described in detail elsewhere [13, 14]. In brief, beta cell function variables were obtained from the OGTT using a model that describes the relationship between insulin secretion and glucose concentration. Characteristic beta cell function variables reported here are as follows: fasting insulin secretion rate (ISR; in pmol min^−1^ m^−2^); insulin output (i.e. the integral of insulin secretion during the 2 h OGTT); beta cell glucose sensitivity (i.e. the mean slope of the dose–response function relating ISR to glucose concentration within the observed glucose range); rate sensitivity, a marker of early insulin release; and potentiation ratio, quantifying a relative augmentation of glucose sensitivity during the OGTT. Insulin sensitivity was calculated using the oral glucose-derived insulin sensitivity index (OGIS), which provides a validated estimate of whole-body glucose clearance (in ml min^−1^ m^−2^) during the insulin-stimulated conditions of the euglycaemic–hyperglycaemic clamp [16]. For the IVGTT, the acute insulin response (AIR) was calculated as the ratio of mean C-peptide to mean glucose increment between 1 min and 8 min following glucose injection. For this analysis, we tested how model-derived measures can aid in type 1 diabetes risk prediction when combined with other key demographic and metabolic variables. In addition, we assessed the impact of age on declining rates of glucose and insulin sensitivity in DPT-1 progressors prior to the onset of stage 3 type 1 diabetes.

### Statistical analysis

Data are presented as mean ± SD or median (IQR). Group comparisons were performed using the Mann–Whitney *U* or Wilcoxon signed-rank test (for unpaired and paired observations, respectively), and the χ^2^ test for categorical variables. ANCOVA was used to compare groups while controlling for age as a covariate. Kaplan–Meier plots were used to compare diabetes-free survival curves by means of the logrank statistic. Cox proportional hazards models were used to estimate HRs (95% CIs). The proportional hazards assumption was confirmed by examining the log cumulative survival plots. *p* values are two sided, and *p*<0.05 was accepted as statistically significant. All analyses were performed using JMP, version 3.1 (SAS Institute, Cary, NC, USA).

## Results

**Fig. 1:**
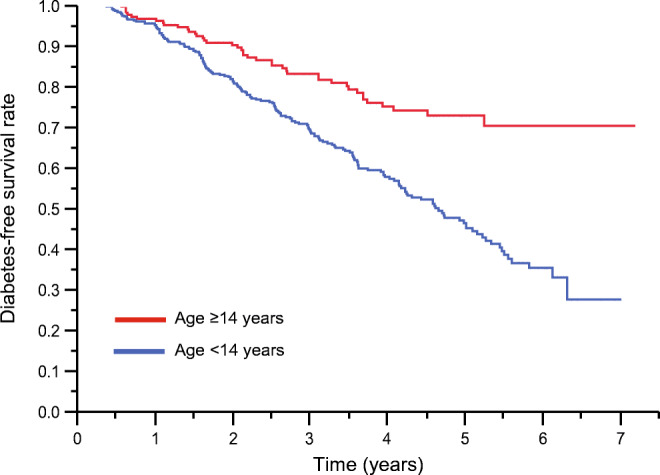
Diabetes-free survival function of study participants by the discriminant age of 14 years (*p*=2.0×10^−7^). Age <14 years, *n*=448; age ≥14 years, *n*=210; 295 prepubertal, 221 peripubertal and 142 adults

Among the 360 participants randomised to receive oral insulin or oral placebo [19], rates of diabetes progression did not differ significantly between the two groups (HR 0.75 [95% CI 0.50, 1.12], *p*=0.16). Likewise, among the 298 participants randomised to receive i.v. injection of insulin or only observation [18], there was no difference between active therapy and no therapy (HR 0.95 [95% CI 0.68, 1.34], *p*=0.78) (electronic supplementary material [ESM] Fig. 1). Therefore, data from the placebo and treatment arms were combined for subsequent analysis. The duration of follow-up was 2.90 (IQR 2.82) years. Among the 658 study participants, 227 (34.4%, or ~12% per year) developed type 1 diabetes. Progressors developed diabetes at a median of 2.24 (IQR 2.13) years, while non-progressors were still free of diabetes at a median 3.41 (IQR 2.80) years of follow-up.

### Impact of age on progression to type 1 diabetes

The impact of age on progression to stage 3 type 1 diabetes was analysed using baseline age as a continuous variable in a univariate Cox regression model. Progression to type 1 diabetes was inversely related to age (HR 0.96 [95% CI 0.94, 0.97] per 1 year, *p*<0.0001). Next, we assessed the relationship between age as a continuous variable and changes in fasting glucose, 2 h glucose and glucose sensitivity. Baseline age was not related to these variables among the entire cohort or in the progressor/non-progressor subgroups (all *p*>0.05). We used χ^2^ serial survival analyses to scan the entire age range of the cohort to identify the highest discriminant age for risk of progression to type 1 diabetes. In this analysis, age 14 years, which divided the cohort roughly into pre- and postpubertal children vs teenagers and adults, doubled the risk of type 1 diabetes development (Fig. 1). This age cut-off was used in subsequent analyses for the following purposes: (1) to compare baseline metabolic measures between progressors and non-progressors aged <14 years and ≥14 years; (2) to test the ability of fasting and model-derived metabolic variables to aid in diabetes risk prediction; and (3) to compare the trajectory of changes in insulin and glucose sensitivity between progressors and non-progressors and between older and younger progressor subgroups.

### Impact of age on baseline metabolic variables

Stratifying by an age cut-off of <14 and ≥14 years, progressors were still younger as compared with non-progressors and had higher baseline prevalence of IGT (Table 1). Sex distribution was similar and autoantibody patterns were not significantly different between any of the groups. As expected, anthropometric measures were higher in both progressors and non-progressors aged ≥14 years compared with progressors and non-progressors aged <14 years.

**Table 1:** Baseline anthropometric and clinical variables of the cohort by outcome and age

Variable	Non-progressors		Progressors		*p*_progr_ value	*p*_age_ value	*p*_g*×*a_ value
	<14 years old	≥14 years old	<14 years old	≥14 years old			
*n*	264	167	184	43			
Sex, male/female, *n*	164/100	83/84	101/83	23/20	NS	NS	NS
Age, years	9.4 (4.9)	19.9 (20.2)	8.8 (4.5)	18.1 (11.9)	0.0436	<0.0001	NS
Height, cm	136 ± 18	172 ± 9	135 ± 19	171 ± 9	NS	<0.0001	NS
Weight, kg	33 ± 13	71 ± 16	34 ± 14	71 ± 16	NS	<0.0001	NS
Body surface area, m^2^	1.13 ± 0.27	1.84 ± 0.23	1.14 ± 0.30	1.85 ± 0.23	NS	<0.0001	NS
BMI, kg/m^2^	17.6 ± 3.5	24.0 ± 4.7	18.0 ± 3.4	24.3 ± 5.4	NS	<0.0001	NS
Glucose tolerance, IGT/NGT, %	5.7	13.8	25.5	46.5	<0.0001	0.0015	NS
ICA, % positive	83.3	82.0	83.7	83.7	NS	NS	NS
ICA512, % positive	44.7	44.9	42.9	41.9	NS	NS	NS
IAA, % high vs normal	78.8	70.7	79.3	79.1	NS	NS	NS
GADA, % positive	67.4	65.9	75.0	60.5	NS	NS	NS

Data are presented as mean ± SD or median (IQR)

*p*_progr_, *p* value for the difference between progressors and non-progressors (regardless of age); *p*_age_, *p* value for the difference between age groups (regardless of progression status); *p*_g*×*a_, *p* value for the progression *×* age interaction, by ANCOVA

GADA, GAD antibody; IAA, insulin autoantibody; NGT, normal glucose tolerance

Fasting glucose was similar between all the groups, whereas 2 h plasma glucose at study entry was 15% higher in progressors (Table 2). Insulin sensitivity was lower in progressors and non-progressors ≥14 years compared with younger progressors and non-progressors. Similarly, fasting C-peptide, fasting insulin, insulin secretory rates and total insulin output were all higher in progressors and non-progressors aged ≥14 years. Glucose sensitivity, which is the mean slope of the dose–response function relating ISRs to glucose concentrations during the OGTT, was reduced by ~46% both in progressors aged <14 years and progressors aged ≥14 years compared with non-progressor groups. Beta cell rate sensitivity, potentiation and insulin sensitivity were similar when comparing progressors with non-progressors.

**Table 2:** Baseline metabolic and functional variables by outcome and age

Variable	Non-progressors		Progressors		*p*_progr_ value	*p*_age_ value	*p*_g*×*a_ value
	<14 years old	≥14 years old	<14 years old	≥14 years old			
*n*	264	167	184	43			
Fasting glucose, mmol/l	5.1 (0.6)	5.1 (0.6)	5.1 (0.6)	5.1 (0.7)	NS	NS	NS
Fasting C-peptide, pmol/l	248 (198)	397 (148)	265 (223)	447 (265)	NS	<0.0001	NS
Fasting insulin, pmol/l	65 (42)	66 (49)	63 (43)	72 (41)	NS	0.0341	NS
Fasting ISR, pmol min^−1^ m^−2^	45 (29)	58 (41)	46 (32)	53 (47)	NS	<0.0001	NS
2 h glucose, mmol/l	5.9 (1.5)	6.0 (2.0)	6.7 (2.0)	7.0 (2.8)	<0.0001	NS	NS
Total insulin output, nmol/m	27.9 (12.0)	29.1 (13.1)	25.3 (13.8)	26.6 (15.0)	0.0006	0.0023	NS
Glucose sensitivity, pmol min^−1^ m^−2^ [mmol/l]^−1^	84 (66)	84 (68)	49 (44)	41 (37)	<0.0001	NS	NS
AIR, pmol/mmol	47.4 (39.6)	54.5 (35.5)	29.2 (25.4)	31.9 (27.7)	<0.0001	0.0342	NS
Rate sensitivity, nmol m^−2^ [mmol/l]^−1^	2.2 (4.2)	2.1 (4.1)	2.4 (3.8)	2.6 (4.1)	NS	NS	NS
Potentiation factor ratio	1.3 (0.7)	1.5 (0.9)	1.3 (0.6)	1.5 (0.6)	NS	0.0017	NS
Insulin sensitivity, ml min^−1^ m^−2^	448 (68)	408 (82)	449 (73)	413 (49)	NS	<0.0001	NS

Data are presented as median (IQR)

*p*_progr_, *p* value for the difference between progressors and non-progressors (regardless of age); *p*_age_, *p* value for the difference between age groups (regardless of progression status); *p*_g*×*a_, *p* value for the progression *×* age interaction, by ANCOVA

### Diabetes risk prediction using age in combination with either fasting or OGTT/IVGTT-derived metabolic variables

**Fig. 2:**
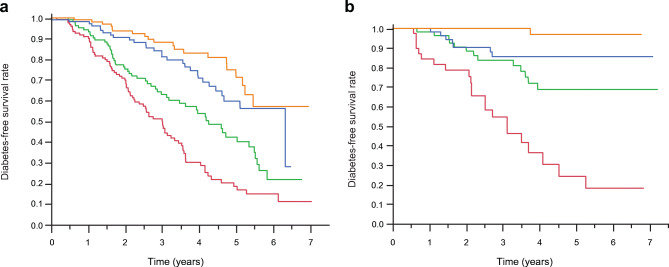
Diabetes-free survival function by sex-specific quartiles of beta cell glucose sensitivity in participants aged <14 years (**a**) (*n*=448, *p*=3.4×10^−15^) or ≥14 years (**b**) (*n*=210, *p*=2.4×10^−12^). Colours indicate glucose sensitivity quartiles (red, green, blue and orange, from the lowest to the highest quartile). Each quartile includes 112 participants aged <14 years and 53 participants aged ≥14 years; progressors, *n*=227; non-progressors *n*=431

**Fig. 3:**
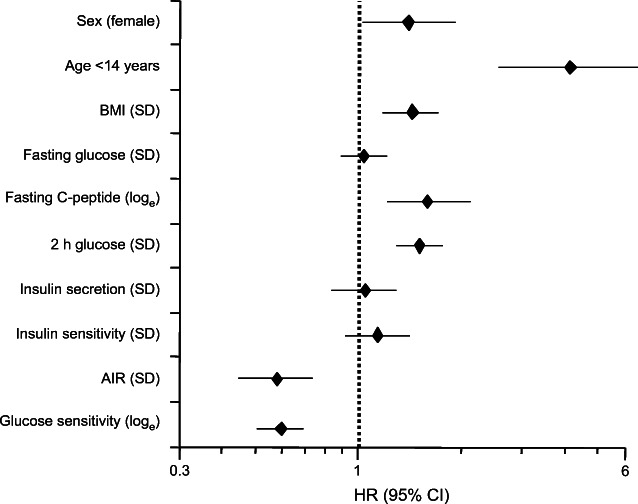
Multivariate Cox proportional hazard model of diabetes progression in the entire cohort (*n*=658). Plots show HR and 95% CI. Insulin secretion represents total insulin output over the 2 h of the OGTT; AIR represents the AIR to i.v. injection of glucose

To identify metabolic variables that were predictive for the development of diabetes, we ran a multivariate Cox model including sex, BMI, fasting plasma glucose, insulin, C-peptide concentrations and fasting ISR. In this model, only age <14 years was a significant predictor of progression to diabetes (HR 3.12 [95% CI 2.04, 4.86]). In contrast, in univariate analysis, sex-specific quartiles of beta cell glucose sensitivity were strongly associated with progression, predicting that at 3 years of follow-up, 50% of the participants in the lowest quartile (median glucose sensitivity 30 [IQR 13] pmol min^−1^ m^−2^ [mmol/l]^−1^) would develop diabetes vs 5% of those in the top quartile (median glucose sensitivity 148 [IQR 63] pmol min^−1^ m^−2^ [mmol/l]^−1^). Notably, the survival curves were evenly graded both in participants aged <14 years and in participants aged ≥14 years (Fig. 2). At follow-up, the insulin secretion/plasma glucose dose–response function was markedly shifted downward and to the right in progressors but not in non-progressors (ESM Fig. 2). A multivariate Cox model including both fasting and OGTT/IVGTT-based variables (Fig. 3) showed that in addition to beta cell glucose sensitivity, age <14 years, fasting C-peptide and 2 h glucose were significant predictors of progression, totalling an AUC of the receiver operating characteristic curve (AUC_ROC_) of 0.81 (*p*=2.5×10^−34^). This was a large improvement over a model using only sex, age, BMI and fasting plasma glucose (AUC_ROC_=0.63, *p*=2.7×10^−6^).

### Time trajectories of changes in insulin and glucose sensitivity

**Fig. 4:**
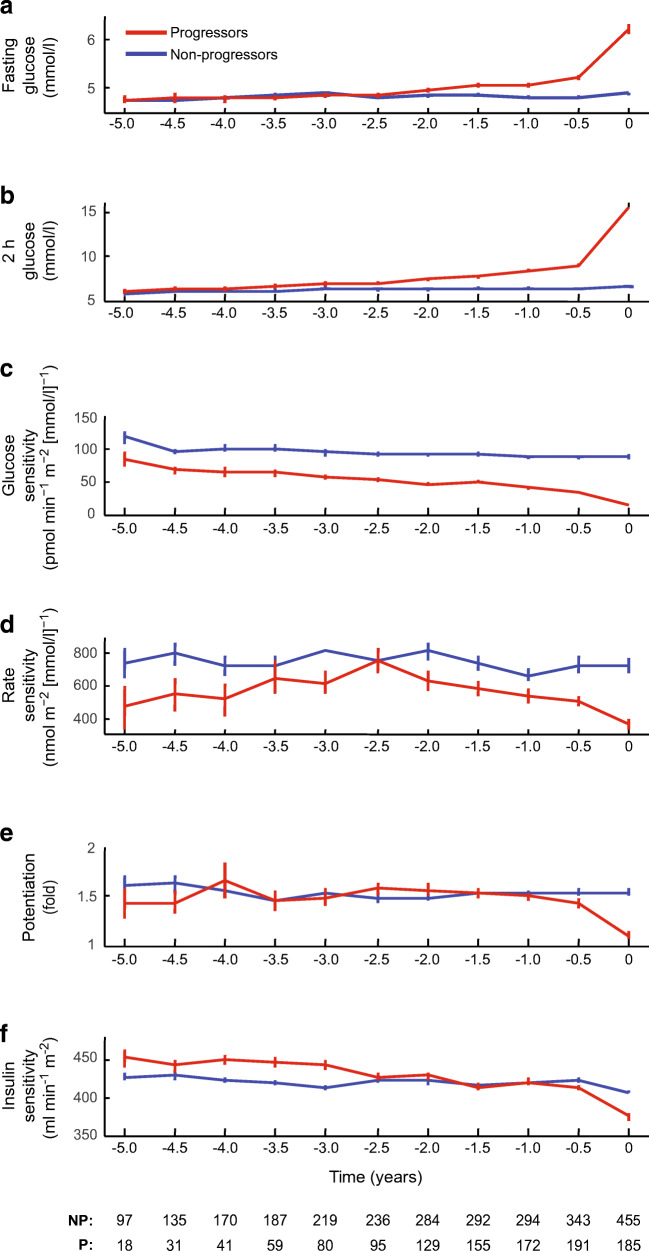
Time course of plasma glucose levels, beta cell function variables and insulin sensitivity by outcome (progressors vs non-progressors). Time 0 is the time of diabetes diagnosis or study end. Numbers of participants are given at the bottom of the figure. NP, non-progressors; P, progressors

The time course of the most relevant metabolic variables was reconstructed using all available OGTTs (*n*=4152). For each variable, the time trajectory was split by outcome (progressors vs non-progressors). As depicted in Fig. 4, in progressors, both fasting and 2 h glucose levels rose rapidly within 0.5–1.0 years before diagnosis as compared with the stable glucose levels of non-progressors. This biphasic pattern was in phase with declines in glucose sensitivity and rate sensitivity, potentiation and insulin sensitivity. Using year −1 as the trajectory inflection point, the rate of decline in glucose sensitivity until year −1 was only slightly faster in progressors than in non-progressors (−9.4 [−27.0] vs −4.0 [−24.5] pmol min^−1^ m^−2^ [mmol/l]^−1^ per year, median [IQR]; *p=*0.03). In contrast, the ‘late’ (year −1 to year 0) rate of decline was approximately fourfold faster in progressors than in non-progressors (−25.8 [−57.2] vs −6.5 [−72.3], median [IQR], *p*<0.0001).

**Fig. 5:**
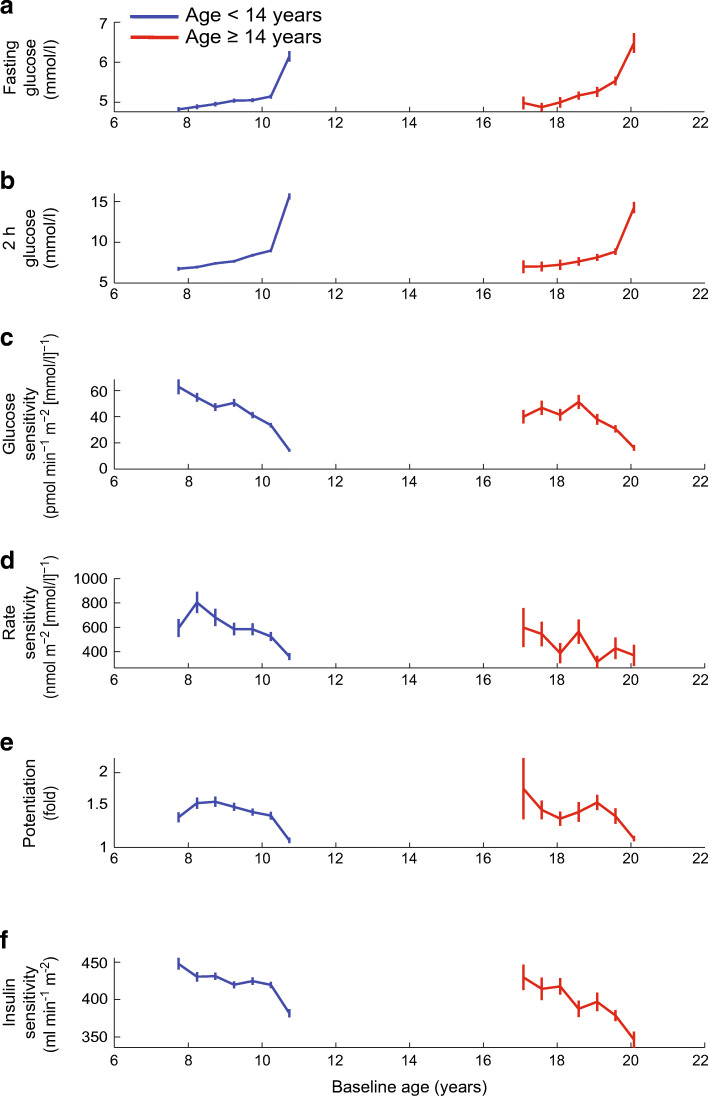
Time course of plasma glucose levels, beta cell function variables and insulin sensitivity by baseline age in progressors aged <14 years (*n*=184) and ≥14 years (*n*=43). Within each age group, trajectories are first aligned to the age of diagnosis (as in Fig. 4). Then, trajectories are shifted by the median group-specific age at diagnosis (last time point is the median age at diagnosis in the group)

Finally, we aimed to compare the trajectories of the change in glucose sensitivity and insulin sensitivity in older and younger progressors. When stratifying by baseline age (Table 3), there were no significant differences in the yearly rate of decline in either glucose sensitivity (−13.7 [21.2] vs −11.9 [21.5] pmol min^−1^ m^−2^ [mmol/l]^−1^, median [IQR], *p*=0.52) or insulin sensitivity (−22 [37] vs −14 [40] ml min^−1^ m^−2^, median [IQR], *p*=0.07) when comparing the progressors aged <14 years with progressors aged ≥14 years. When focusing on early and late time segments, the rate of decline in glucose sensitivity until year −1 was similar in older vs younger progressors (−4.1 [−24.0] vs −6.7 [−26.3] pmol min^−1^ m^−2^ [mmol/l]^−1^ per year, median [IQR], *p*>0.05). In addition, the ‘late’ (year −1 to year 0) rate of decline did not significantly differ (−14.1 [−55.8] vs −19.8 [−54.2] pmol min^−1^ m^−2^ [mmol/l]^−1^ per year, median [IQR], *p*>0.05) between older and younger progressors. When the trajectories of the progressors in the two age groups were plotted against their respective baseline age (Fig. 5), the patterns of change of the physiological variables were remarkably similar between younger and older participants (confirming the data presented in Table 3). Overall, these data indicate that the impact of baseline age is to shorten the time at which the physiological variables start to change (i.e. the inflection point of the biphasic time course), with similar rates of change in both beta cell glucose sensitivity and insulin sensitivity observed during this period of active progression.

**Table 3:** Yearly rate of change in metabolic variables between baseline and follow-up in progressors and non-progressors by age group

Variable	Non-progressors		Progressors		*p*_*progr*_ value	*p*_*age*_ value	*p*_*g×a*_ value
	<14 years old	≥14 years old	<14 years old	≥14 years old			
*n*	264	167	184	43			
Fasting glucose, mmol/l	0.02 (0.22)	0.05 (0.34)	0.29 (0.57)	0.38 (0.65)	<0.0001	NS	NS
Fasting ISR, pmol min^−1^ m^−2^	6.1 (12.2)	1.3 (9.8)	5.1 (16.0)	−0.2 (21.3)	NS	NS	NS
2 h glucose, mmol/l	0.13 (0.67)	0.10 (0.77)	2.50 (6.80)	1.73 (5.09)	<0.0001	NS	NS
Total insulin output, nmol/m^2^	1.4 (3.8)	0.3 (4.1)	−1.9 (5.7)	−2.6 (6.4)	<0.0001	NS	NS
Glucose sensitivity, pmol min^−1^ m^−2^ [mmol/l]^−1^	−2.7 (20.6)	−3.4 (29.3)	−13.7 (21.2)	−11.9 (21.5)	<0.0001	NS	NS
Insulin sensitivity, ml min^−1^ m^−2^	−8 (23)	−4 (25)	−22 (37)	−14 (40)	<0.0001	NS	NS
Rate sensitivity, nmol m^−2^ [mmol/l]^−1^	0 (384)	−28 (353)	−81 (354)	−72 (305)	<0.0004	NS	NS
Potentiation factor ratio	0.01 (0.31)	−0.01 (0.19)	−0.09 (0.33)	−0.12 (0.42)	0.0071	NS	NS

Data are presented as median (IQR)

*p*_progr_, *p* value for the difference between progressors and non-progressors (regardless of age); *p*_age_, *p* value for the difference between age groups (regardless of progression status); *p*_g×a_, *p* value for the progression × age interaction, by ANCOVA

**Fig. 6:**
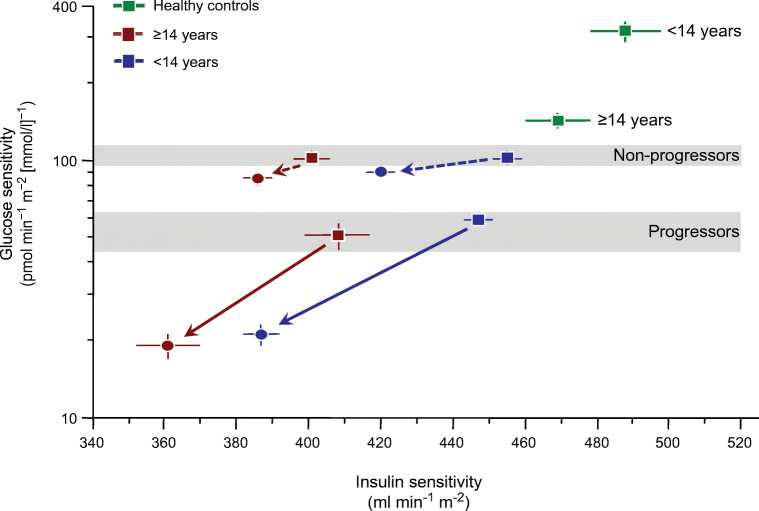
Scattergram of glucose sensitivity against insulin sensitivity in progressors and non-progressors by age <14 or ≥14 years (progressors aged <14 years, *n*=184; progressors aged ≥14 years, *n*=43; non-progressors aged <14 years, *n*=264; non-progressors aged ≥14 years, *n*=167). Historical data from healthy control groups of participants aged ≥14 years (*n*=72; age 30 ± 2 years, BMI 24.2 ± 5.0 kg/m^2^, mean ± SD) and younger individuals (*n*=68; age 9 ± 2 years, BMI 23.6 ± 1.1 kg/m^2^, mean ± SD) are also plotted. Squares, baseline values; circles, follow-up values; dashed arrows connect non-progressors; solid arrows connect progressors. Plots are mean ± SEM

A graphical summary of the overall changes in glucose and insulin sensitivity is shown in Fig. 6, which includes data from a group of healthy children under 14 years of age and another group of participants over the age of 14 years, each sex- and BMI-matched to the corresponding age group of our DPT-1 participants. On this graph, younger participants lie to the right of older participants on the scale of insulin sensitivity whether they are progressors, non-progressors or healthy control individuals. Furthermore, healthy participants of either age group have far better glucose sensitivity and insulin sensitivity than either DPT-1 progressors or non-progressors. Progressors start with worse glucose sensitivity than non-progressors and lose more glucose and insulin sensitivity over time. However, among progressors aged <14 years and progressors aged ≥14 years, the slopes of decline in glucose sensitivity and insulin sensitivity were not different, suggesting that age does not influence patterns of metabolic decline in autoantibody-positive individuals followed longitudinally until stage 3 type 1 diabetes onset.

## Discussion

We assessed the impact of age on patterns of metabolic progression from early stage type 1 diabetes to stage 3 diabetes onset among autoantibody-positive individuals followed longitudinally in the DPT-1 study. We applied a previously validated mathematical model to define changes in measures of beta cell function and insulin sensitivity in 658 individuals who completed 4152 OGTTs over an extended period of follow-up. The analysis confirmed previous findings showing that risk of progression to type 1 diabetes is inversely related to age [4]. In contrast, age did not affect the rate of change of declining beta cell glucose sensitivity or insulin sensitivity in individuals who progressed to a clinical diagnosis of type 1 diabetes. Declines in both beta cell glucose sensitivity and insulin sensitivity were similar between progressors aged <14 years and progressors aged ≥14 years. Taken together, these data suggest that individuals who are classified as high risk at a younger age have a higher probability of developing diabetes but the evolution towards diabetes is similar to that of older individuals, being characterised by a relatively stable time course until an inflection point (occurring earlier in younger individuals), after which a rapid deterioration occurs (Fig. 5).

Some but not all studies suggest that age may influence the rate of C-peptide decline after the clinical diagnosis of type 1 diabetes (i.e*.* stage 3 disease onset). In individuals followed for 2 years post diagnosis in the placebo arms of several TrialNet studies, the slope of decline in the C-peptide AUC values from mixed meal tolerance tests was increased in children compared with adults [5]. In contrast, analysis of two UK cohorts and a Swedish cohort found similar rates of C-peptide decline among old and young study participants [7, 8]. Despite these discrepancies, in very-long-duration disease, detectable C-peptide is more likely to be observed in those with an older age at diagnosis [9–12].

To test whether age influenced patterns of metabolic progression prior to the onset of stage 3 type 1 diabetes, we utilised a mathematical model that reconstructs insulin sensitivity and endogenous insulin secretion from C-peptide kinetics [20] and resolves three main modes of beta cell function, namely the glucose dose–response, early insulin secretion, and potentiation [13–17]. In our cohort, the highest discriminant age for diabetes risk was 14 years, which divided the cohort into pre- and postpubertal children vs teenagers and adults. The risk of type 1 diabetes was doubled in progressors aged <14 years compared with progressors aged ≥14 years. We found that older DPT-1 participants (i.e. both progressors and non-progressors aged ≥14 years) were more insulin resistant at baseline and throughout the monitoring period. We did not detect baseline differences in insulin sensitivity between progressors and non-progressors, a finding that differs from results of an analysis of the Melbourne Pre-Diabetes Family Study and the ENDIT study cohort [21, 22]. These differences could be related to differences in demographic characteristics between the cohorts or could be secondary to the methods used to calculate insulin sensitivity.

Nonetheless, during follow-up, insulin sensitivity declined in both young and old progressors. However, the annualised rate of decline did not differ between progressors <14 years and progressors ≥14 years. On average, beta cell glucose sensitivity at study entry was ~46% lower in progressors than in non-progressors and was impaired to a similar extent in both age groups. Consistent with this, sex-specific quartiles of beta cell glucose sensitivity were strongly associated with progression to type 1 diabetes, suggesting that this measure identifies high-risk individuals across the age span. Similar to insulin sensitivity, the rates of annual decline in beta cell glucose sensitivity were not different when comparing progressors aged <14 years with progressors aged ≥14 years. We analysed the rates of decline in both glucose and insulin sensitivity from study entry to year −1 and from year −1 to diabetes onset. When analysing these time segments separately, there were no significant differences in either the rate of decline in insulin sensitivity or the rate of decline in beta cell glucose sensitivity between young and old participants.

Our results differ somewhat from a recent analysis performed on a small subset of 80 individuals followed in the TrialNet Pathway to Prevention cohort who underwent OGTTs before and after stage 3 type 1 diabetes onset [23]. In that analysis, younger progressors exhibited a faster rate of decline in C-peptide AUC in the 6 months before and after diabetes diagnosis, with a similar slope of decline across this peridiagnostic period. While C-peptide AUC has been the accepted endpoint for trials performed at stage 3 onset, this measure provides incomplete information about beta cell function as compared with beta cell glucose sensitivity, because it fails to account for insulin secretion in response to prevailing blood glucose levels [24, 25]. In addition, the study did not examine C-peptide indices prior to the −6 month time period and did not assess changes in insulin sensitivity, information that is provided by our analysis.

There are limitations to the present analysis. First, this is a secondary analysis of existing clinical trial data, and a post hoc power analysis was not performed. Second, formal testing of the non-linear dependence of risk on age was not attempted. We assessed metabolic trajectories using dichotomous age stratification and age 14 years as a cut-off. The age cut-off of 14 years was selected based on the observation that risk of diabetes more than doubled in children <14 years of age compared with those ≥14 years of age. However, we caution against the adoption of this age cut-off without further validation in another cohort. Finally, insulin sensitivity was calculated using a proxy (OGIS) rather than euglycaemic insulin clamps.

Notwithstanding these limitations, our findings could have important implications for the implementation of disease-modifying therapy in early stage type 1 diabetes. Recent results from prevention trials using teplizumab and oral insulin initiated in stage 1 and 2 type 1 diabetes suggest that autoantibody-positive individuals with accelerated, active disease and paradoxically worse beta cell function may be primed to respond better to a disease-modifying intervention [26, 27]. While children have historically responded more robustly to immune interventions initiated after stage 3 onset, a prespecified subgroup analysis of the teplizumab prevention trial did not indicate a similar age effect in individuals in stage 2 disease who were treated with a single 14 day course of drug [26, 28]. These learnings are now being leveraged to design criteria for clinical trial inclusion based on the presence of high-risk metabolic markers, which provide information on risk of developing diabetes within a certain time horizon and are being leveraged to define periods of active, accelerated disease [29]. Defining whether there are differences in metabolic patterns of progression among individuals with autoantibody positivity across a diverse range of ages is a necessary precursor for these efforts.

## Supplementary information

ESM (PDF 1172 kb)

## Data Availability

Data from this study are available from the corresponding author upon reasonable request for the purposes of a collaborative research project.

## Abbreviations

AIR: Acute insulin response
DPT-1: Diabetes Prevention Trial–Type 1
ICA: Islet cell antibody
IGT: Impaired glucose tolerance
ISR: Insulin secretion rate
OGIS: Oral glucose-derived insulin sensitivity index
