# Supplementary material for: The effect of age on longitudinal measures of beta cell function and insulin sensitivity during the progression of early stage type 1 diabetes
Source: Diabetologia. 2022 Dec 2;66(3):508–19. doi: 10.1007/s00125-022-05836-w (PMC9716154; doi:10.1007/s00125-022-05836-w)
Supplement: Supplementary file 1 — (PDF 1172 kb) [file 125_2022_5836_MOESM1_ESM.pdf]

Electronic supplementary material

The effect of age on longitudinal measures of beta cell function and insulin sensitivity during the progression of early stage type 1 diabetes

Ele Ferrannini, Andrea Mari, Gabriela S.F. Monaco, Jay S. Skyler, Carmella Evans-Molina

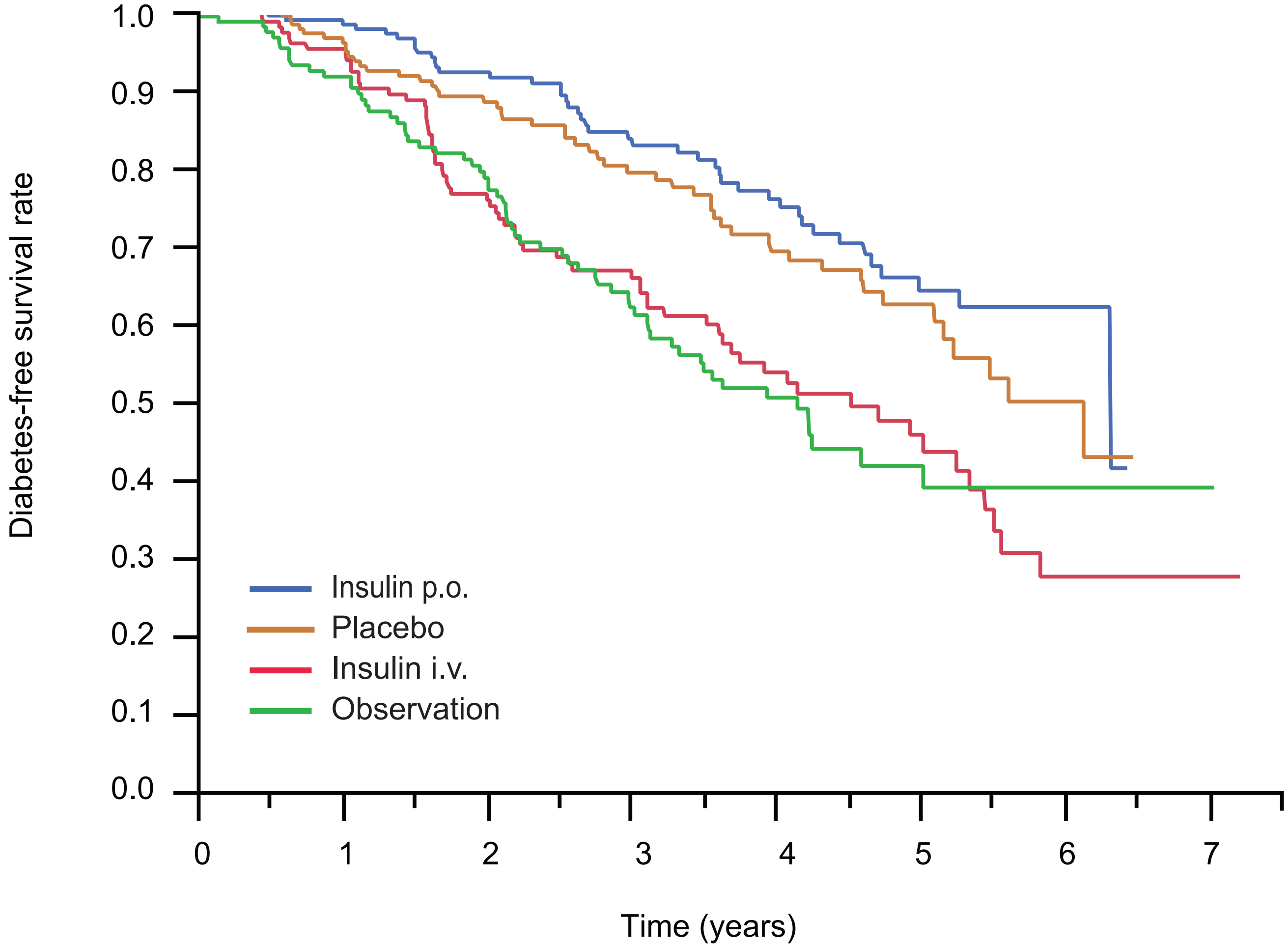

ESM Figure 1 – Diabetes-free survival function in 360 subjects randomised to receive oral insulin (*insulin p.o.*) or oral placebo (*plb*) and in 298 subjects randomised to receive intravenous insulin (*insulin i.v.*) or only observation (*obs*).

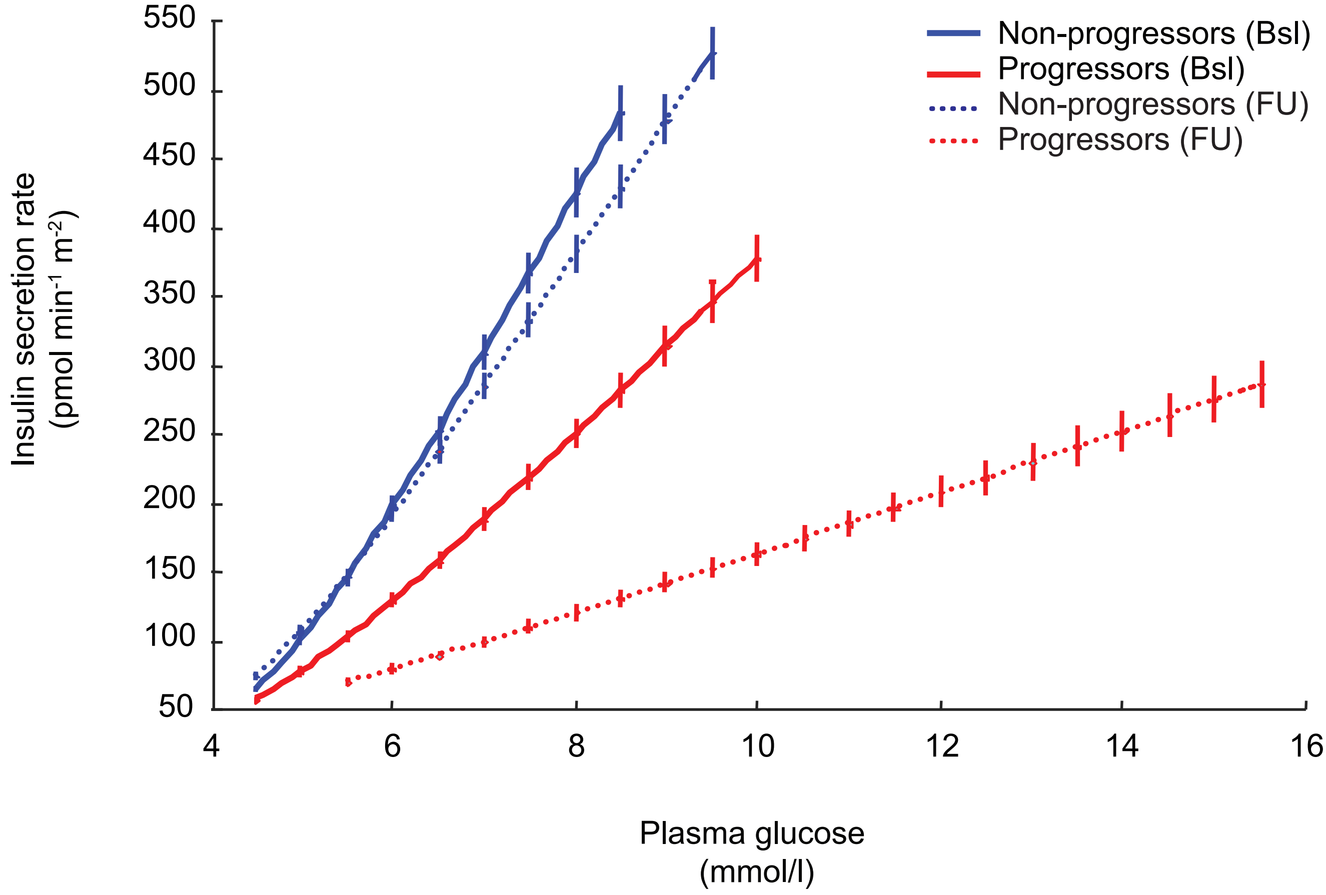

ESM Figure 2 – Insulin secretion-plasma glucose dose-response function in progressors (*n*= 227) and non-progressors (*n*= 431). Bsl = baseline; FU = follow up. Plots are mean ± SEM
